# Response of seaward-migrating European eel (*Anguilla anguilla*) to manipulated flow fields

**DOI:** 10.1098/rspb.2015.1098

**Published:** 2015-07-22

**Authors:** Adam T. Piper, Costantino Manes, Fabio Siniscalchi, Andrea Marion, Rosalind M. Wright, Paul S. Kemp

**Affiliations:** 1 International Centre for Ecohydraulics Research, Faculty of Engineering and the Environment, University of Southampton, Southampton SO17 1BJ, UK; 2 Department of Industrial Engineering, University of Padua, via Marzolo 9, Padova 35131, Italy; 3 Environment Agency, Rivers House, Threshelfords Business Park, Inworth Road, Feering CO5 9SE, UK

**Keywords:** behavioural fish guidance, hydrodynamics, hydropower, acoustic telemetry, computational fluid dynamics, ecohydraulics

## Abstract

Anthropogenic structures (e.g. weirs and dams) fragment river networks and restrict the movement of migratory fish. Poor understanding of behavioural response to hydrodynamic cues at structures currently limits the development of effective barrier mitigation measures. This study aimed to assess the effect of flow constriction and associated flow patterns on eel behaviour during downstream migration. In a field experiment, we tracked the movements of 40 tagged adult European eels (*Anguilla anguilla*) through the forebay of a redundant hydropower intake under two manipulated hydrodynamic treatments. Interrogation of fish trajectories in relation to measured and modelled water velocities provided new insights into behaviour, fundamental for developing passage technologies for this endangered species. Eels rarely followed direct routes through the site. Initially, fish aligned with streamlines near the channel banks and approached the intake semi-passively. A switch to more energetically costly avoidance behaviours occurred on encountering constricted flow, prior to physical contact with structures. Under high water velocity gradients, fish then tended to escape rapidly back upstream, whereas exploratory ‘search’ behaviour was common when acceleration was low. This study highlights the importance of hydrodynamics in informing eel behaviour. This offers potential to develop behavioural guidance, improve fish passage solutions and enhance traditional physical screening.

## 1. Introduction

Globally, freshwater ecosystems are the most anthropogenically impacted, in part due to a loss of connectivity caused by infrastructure such as weirs, dams and other impediments [1–3]. In-channel structures may inhibit or prevent the movement of aquatic biota [4], causing population decline, or even extirpation [5]. For fish, physical barriers obstruct dispersal and migration between habitats required for different ontogenetic stages, and thus disrupt the life cycle [6,7]. River infrastructure, such as hydropower and pumping facilities, can also cause direct injury and mortality to fish that pass through them due to blade strike, cavitation and grinding [8,9]. Further, migratory delay at structures may increase susceptibility to predation, parasites and infectious diseases, and impose energetic costs [7,10].

Despite centuries of efforts to restore and maintain connectivity for fish (typically by providing fish passes), effective solutions remain elusive under many scenarios [11–13]. The development of effective fish passage depends on fundamental knowledge of swimming capabilities, which has received much attention [14], with a historical bias towards salmonids [15,16]. However, this must be combined with an understanding of behavioural response to environmental stimuli [4,17], both those that attract and repel fish [18]. This knowledge is currently lacking for many species [12,18] and there is insufficient understanding of the transferability of salmonid research to other fish. As fish move with the flow during their downstream migration, it is expected that behavioural response will be more influential than swimming capability, compared with upstream-moving migrants, for which both components play an important role [18].

Fish gain information about their spatial location from multiple stimuli [19]. The discriminability of a specific stimulus and the subsequent response elicited is dependent on both its absolute and relative magnitude in comparison with background noise [20]. Further, discriminability differs among species [9] and ontogenetic stage [7], and with motivation [21], behavioural bias [20], prior experience, learning and habituation [22]. In the complex environments encountered at river infrastructure, hydrodynamic factors probably constitute the dominant cues that inform fine-scale navigation and route selection [23,24].

On a broad scale, the high proportion of river flow diverted through water intakes (such as at hydropower plants or other abstraction points) presents a strong directional cue to downstream-migrating fish that encounter them [15,25]. Fish react to localized changes in flow field characteristics, including turbulence [26,27] and spatial velocity gradient [28], using the lateral line to detect flow strength and direction [29,30] and the otolith of the inner ear to detect whole-body acceleration, deceleration and gravitation [31,32]. The rapid acceleration of flow at constrictions such as at intake channels and downstream fish passage facilities (hereafter referred to as bypasses) can elicit rejection behaviour among downstream-migrating juvenile salmonids [33–35]. Hydrodynamics may also explain observed rejection at river structures for other species [36–38], although understanding is limited for non-salmonids [12,39].

The severe decline of the critically endangered European eel (*Anguilla anguilla*) has in part been attributed to delayed or blocked seaward migration of escaping adults (silver eels) at river infrastructure [40]. Eels suffer high rates of injury and mortality at pumps and hydropower turbines (typically 15–38% per turbine encountered [41,42]), and are susceptible to impingement at exclusion screens [37,43]. For the few downstream passage solutions trialled for eels, effectiveness is highly variable but generally low [44–46]. Adult eels tend to follow routes of bulk flow [36,47], but on encountering structures display exploratory behaviour and make multiple approaches before passing [46,48,49]. The resolution at which both hydrodynamics and fish migratory paths have been quantified in the field is generally insufficient to determine the relative roles of localized variation in the flow field and physical contact with structures in eliciting specific behaviours [45,49,50]. In common with other diadromous fish species, there has been a historical focus on physical as opposed to behavioural exclusion or guidance for eels. Flume-based studies report eel rejection behaviour after contact with structures such as screens [51,52], leading to the view that, compared with salmonids, adult eels are less sensitive to changes in velocity [18]. This thigmotactic propensity of eels increases the probability of impingement and injury at screens, emphasizing the urgent need to find alternative mitigation solutions.

Given the likely role of riverine barriers in the decline of the European eel and our current lack of knowledge about their response at structures, this study aimed to assess the effect of flow fields on the behaviour of adults during downstream migration. Using acoustic telemetry that enabled near-continuous tracking of fish at sub-metre accuracy, combined with three-dimensional hydrodynamic measurement techniques and computational fluid dynamics (CFD) modelling, a field experiment was conducted to quantify eel responses to manipulated flow fields under two treatments: (1) unrestricted flow with low water acceleration (unrestricted low, UL), and (2) constricted flow with high water acceleration (constricted high, CH). We quantified: (i) behavioural response to flow fields to investigate how hydrodynamics influence eel behaviour, and (ii) the impact of flow fields on swim path characteristics including eel route choice, residence time and track length as indicators of passage efficiency and energetic cost.

## 2. Material and methods

### (a) Site description and experimental set-up

**Figure 1.**
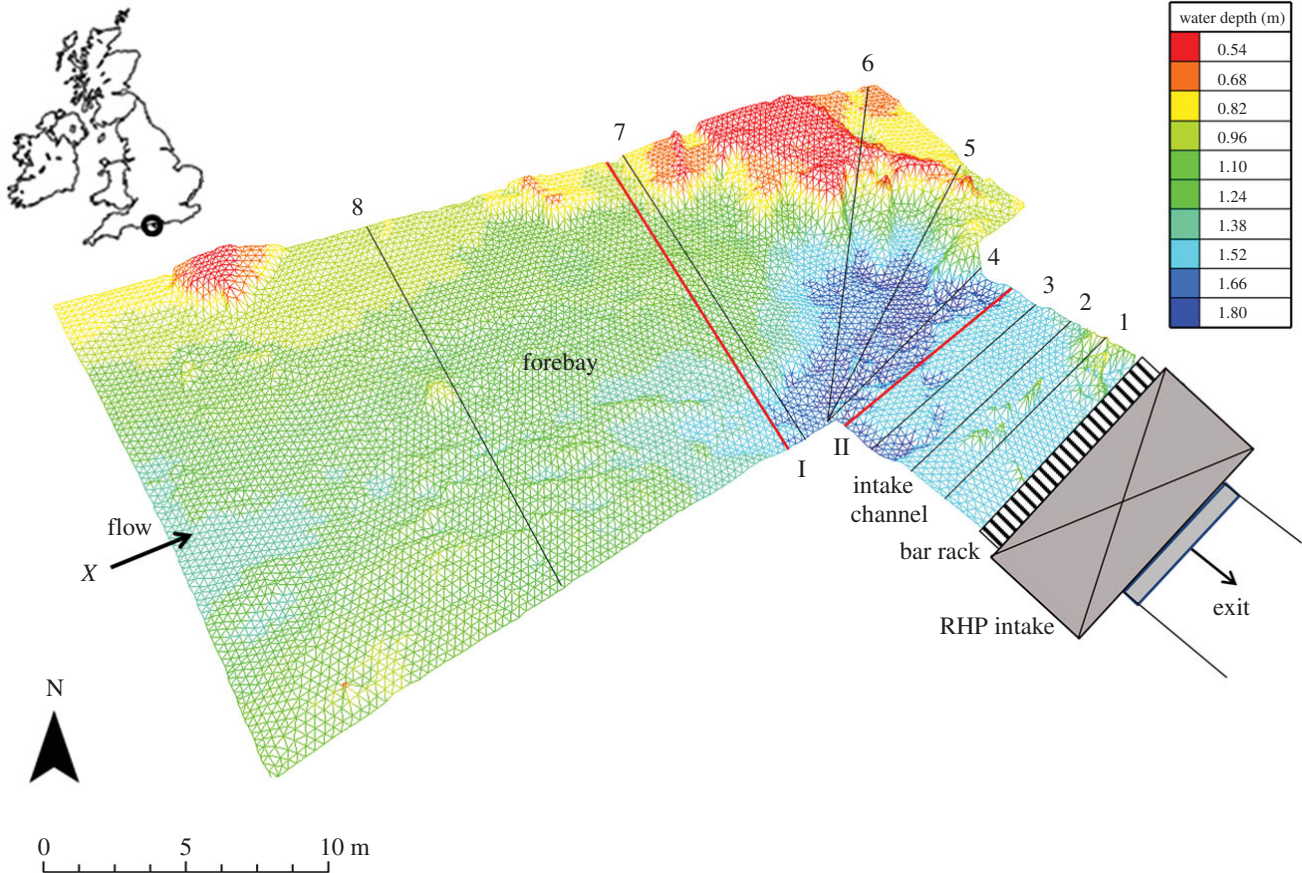
Bathymetry within the forebay and intake channel at a RHP facility on the River Stour, Dorset, UK, during the study period, November 2010. Black lines indicate transects (1–8) along which water velocities were recorded; red lines indicate PIT antennae (I and II). *X* denotes the fish release point.

The study was conducted in the forebay upstream of a redundant hydropower (RHP) facility at Longham on the River Stour, Dorset, UK (50°43′28.26″ N, 1°44′16.57″ W). The forebay channel narrows from 17.0 to 12.2 m width, at which point flow is diverted down an intake channel (7.6 m, width) oriented 90° to the forebay (figure 1). The RHP originally housed two turbines but ceased operating during the 1970s.

The intake channel was manipulated to generate two hydrodynamic treatments: (1) UL and (2) CH. A sloping bar rack (7.6 m width, 55° angle, 58 mm vertical bar spacing) extended the full depth of the water column at the RHP intake. In the UL treatment, flow through the bar rack was relatively uniform across the full width of the intake channel. In the CH treatment, the flow was constricted by 66% by wooden boards placed on the upstream face of the bar rack to leave a full-depth opening in the centre channel. Undershot sluice gates 5 m downstream of the bar rack were manipulated to ensure equal flow passed under both treatments (6.28 ± 0.2 m^3^ s^−1^). Natural fluctuations in water level were controlled by diverting flow via a radial weir directly upstream of the study site.

Hydrodynamics in the two treatments were quantified using water velocity and bathymetry measurements collected with a raft-mounted downward-looking acoustic Doppler current profiler (ADCP; RiverSurveyor ADP M9, SonTek, San Diego, CA, USA), and used to inform and calibrate a two-dimensional CFD model of water velocity within the study site. The ADCP was pulled along tensioned guide wires at eight transect locations (figure 1), repeated before each trial. Data were visually inspected using RiverSurveyor Live v. 3.01 and exported to MATLAB (R2010a, Mathworks, Natick, MA, USA) for removal of outliers (after Dinehart & Burau [53]) and calculation of depth-averaged velocities for each measured velocity profile. Data from the most upstream transect were used to calculate total channel flow. Site bathymetry (figure 1) was mapped using the ADCP with a 0.5 MHz vertical acoustic beam [53].

**Figure 2.**
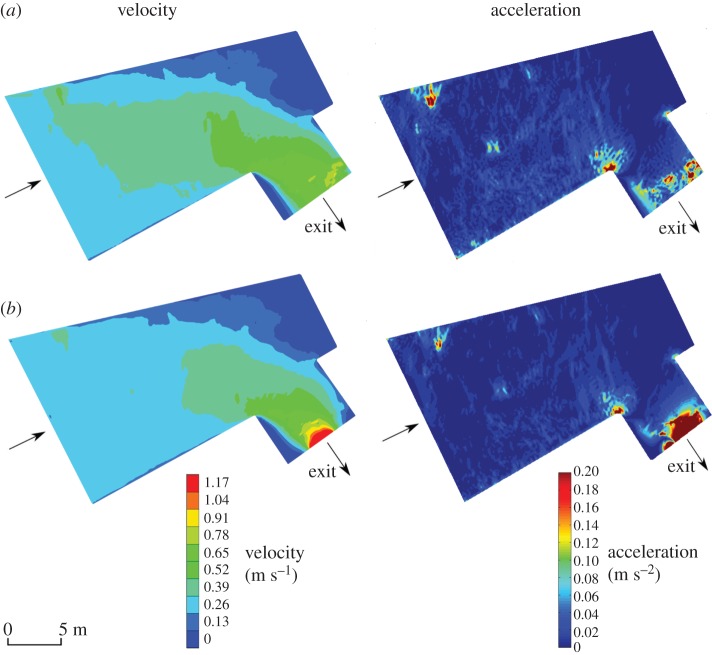
Modelled depth-averaged water velocity and acceleration under two hydrodynamic treatments: (*a*) unrestricted flow with low water acceleration (UL) and (*b*) constricted flow with high water acceleration (CH). Arrows indicate flow direction.

To compensate for limitations of ADCP flow mapping (e.g. limited spatial resolution and poor accuracy at domain boundaries), a two-dimensional hydrodynamic model (TELEMAC-2D) [54] was constructed using ADCP-derived empirical data. The flow domain was discretized with a mesh of two-dimensional finite triangular elements (0.005–0.25 m dependent on resolution required to adequately capture gradients), and in each node the code solved the depth-averaged free surface flow equations (de Saint-Venant equations) to obtain water depth and depth-averaged velocity components. Boundary conditions were assigned to the nodes at the domain border. A fixed flow rate and water elevation were allocated at the domain entrance and exit, respectively, and the remaining boundary was assumed to be impermeable solid banks. After calibration using ADCP measurements with control parameters (e.g. bed roughness coefficient) adjusted as necessary, the model reproduced the flow field reasonably well in terms of both depth-averaged mean velocities and flow depth. The simulated depth-averaged velocity fields provide confidence on the effectiveness of the chosen treatments (figure 2).

Flow accelerations were estimated from the depth-averaged velocities obtained from the hydrodynamic model in which the velocity vector was defined as *U*(*u*, *v*), where *u* and *v* are the velocity components along *x* and *y* (geographical east and north), respectively. The module of the total acceleration at each point was estimated as
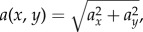
where *a*_*x*_ = *u*(∂*u*/∂*x*) + *v*(∂*u*/∂*y*) and *a*_*y*_ = *u*(∂*v*/∂*x*) + *v*(∂*v*/∂*y*) are the components of the acceleration *a* along *x* and *y*, respectively.

### (b) Fish telemetry

Actively migrating adult eels (*n* = 40) were tracked using acoustic and passive integrated transponder (PIT) telemetry. Eight hydrophones (300 kHz) around the perimeter of the study site and a receiver (HTI, Model 290, Hydroacoustic Technology Inc., Seattle, WA, USA) logged all acoustic tag detections. Due to the shallow water depths, it was not possible to accurately determine tag position in the *z*-dimension. PIT telemetry was used to quantify swim depth at two swim-over antennae and receiver stations (Model LF-HDX-RFID Oregon, Portland, OR, USA), which covered the full channel width in the forebay (14 m length, 0.5 m width) and intake channel (7.5 m length, 0.5 m width) (figure 1).

On the night preceding each trial, actively migrating silver eels were captured at a rack trap downstream of the RHP facility. Fish were maintained in within-river perforated plastic holding barrels (220 l) for a maximum of 8 h before being individually anaesthetized (benzocaine 0.2 g l^−1^), weighed (wet weight, W, g) and measured (total length, TL, mm). The length of the left pectoral fin (mm) and maximum vertical and horizontal left eye diameter (mm) were used to determine the degree of sexual maturation or ‘silvering’. The first five eels considered migratory (following methods of Pankhurst [55] and Durif *et al*. [56]) were selected for tagging each night. An acoustic (HTI model 795G, 11 mm diameter, 25 mm length, 4.5 g mass in air, 300 kHz, 0.7–1.3 s transmission rate) and PIT (Texas Instruments, HDX, 3.65 mm diameter, 32 mm length, 0.8 g mass in air) tag were surgically implanted into the peritoneal cavity of each individual following methods similar to Baras & Jeandrain [57] and carried out under a UK Home Office licence. Tagged eels ranged from 639 to 921 mm TL, 566 to 1207 g W, with mean Ocular Index 9.1 (range 7.5–13.5) and mean Fin Index 4.9 (range 4.3–5.8).

After recovery, eels were transferred to a perforated holding barrel 3 m upstream of the site and held for 10–12 h. The barrel was tethered in the channel centre to reduce bias in route choice and the lid removed at 20.00 (in darkness) from the bank using a rope and pulley system to minimize disturbance and to enable the eels to leave volitionally.

Five eels were released and tracked through the site per trial (four replicates, yielding 20 eels per treatment). Test treatments were alternated to reduce temporal bias (eight trial nights over a 16 night period). Range-testing using known tag locations demonstrated a minimum accuracy and precision of less than 0.5 m within the hydrophone array. Tag detection at both PIT antennae was consistent (98% and more than 99% at antennae 1 and 2, respectively) for depths of less than 0.2 m.

### (c) Data analysis

Acoustic tag detections were processed using MarkTag v. 5 and AcousticTag v. 5 (Hydroacoustic Technology Inc.), and PIT data were examined for detections when eel tracks intersected antenna locations.

To determine whether treatment induced a behavioural response, tracks were overlaid on maps of flow streamlines in MATLAB. A theoretical boundary was imposed at the point where streamlines began to distort upstream of the bend leading to the intake channel (flow distortion boundary). The distance between two adjacent streamlines was set to 1 m (at the entrance), which is comparable with the uncertainty of the telemetry positioning. Tracks were visually assessed and a set of numerical rules devised to determine when trajectories deviated from the streamlines. These deviations, termed ‘behavioural switch points', were defined as the first point at which a downstream-moving fish exhibited a turn angle of 90–180° (i.e. deviated from the predominate flow direction) and proceeded in the new direction for a minimum of 3 m. Mann–Whitney *U*-tests were used to test for a treatment effect on water velocity and acceleration at the point of behavioural switch.

Based on assessment of trajectories immediately after a behavioural switch, individuals were assigned to one of two categories:

*Rejection*: when downstream-moving fish abruptly switched from negative to positive rheotaxis and moved in a counter streamwise direction for a distance greater than 3 m.

*Exploratory behaviour*: when downstream-moving fish switched from negative rheotaxis to exhibit lateral movements of greater than 3 m length perpendicular to streamwise flow and encompassing more than two turns.

To quantify the effect of behaviours on the speed and efficiency of migration through the site, the following metrics were calculated for each fish: *residence time* (duration between first and last detection before passage through the bar rack), *mean speed over ground* (m s^−1^) and *track length* (m). Movement metrics among treatment groups were compared using *t*-test and Mann–Whitney *U*-test where the assumptions of parametric analysis were not met. Where trajectories were aligned with streamlines, the velocity of fish over ground (m s^−1^) was calculated using the difference in fish position every 5 s compared with mean water velocity in the streamline (m s^−1^). As the study focus was primarily on fish behaviour during movement through the site, near stationary points (values below 0.02 ms^−1^) were eliminated from the dataset.

Trajectory analyses were carried out using a combination of ArcMap (v. 10, ESRI, Redlands, CA, USA), Geospatial Modelling Environment v. 6.0 [58] and MATLAB. R v. 3.0.0 [59] was used for all statistical analyses.

## 3. Results

Of the 40 fish released under the two treatments, three swam upstream shortly after release and did not re-enter the study area; these were omitted. The remaining 37 individuals passed through the RHP intake. There was no indication from swim tracks that eels were impinged on the bar rack during passage (i.e. were not stationary at this structure) under either treatment. Residence time was highly variable and ranged from 2.9 to 58.7 min (median, 10.25 min) across all fish, with no treatment effect (Mann–Whitney *U* = 1.0, *p* = 0.33).

**Figure 3.**
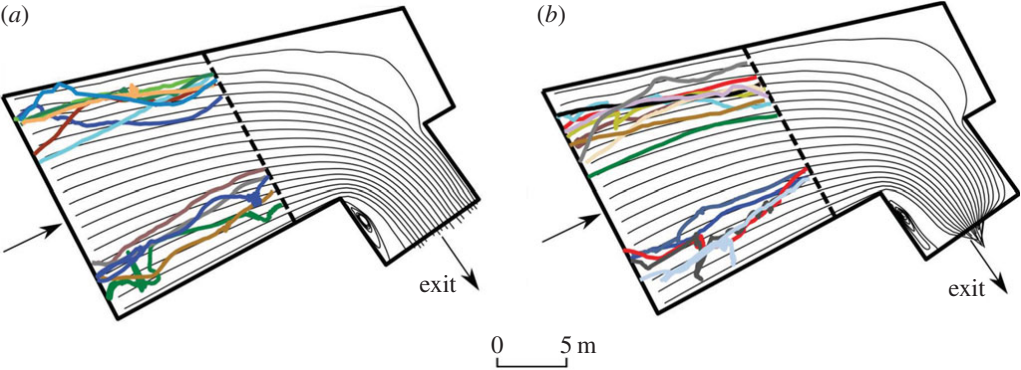
Trajectories of downstream-migrating European eels that aligned with modelled streamlines (73% of fish; 27 out of 37 that passed RHP) in the forebay of a RHP plant on the River Stour, Dorset, UK. Flow was manipulated to create two hydrodynamic treatments: (*a*) unrestricted flow with low water acceleration (UL) and (*b*) constricted flow with high water acceleration (CH). The dashed line indicates a theoretical boundary after which streamlines distorted upstream of the intake channel. Arrows indicate flow direction.

Upstream of the flow distortion boundary, the majority of fish (73%, 27 out of 37) under both treatments followed trajectories reasonably well aligned with streamlines. The preferred routes were along both sides of the channel (figure 3). The mean ratio of eel ground speed to water velocity in streamlines was 0.78 (±0.49 s.d.) in this upstream part of the domain.

As downstream-moving individuals approached the 90° bend at the entrance to the intake channel, trajectories generally became more erratic and switches in behaviour were apparent for 35 out of the 37 eels that reached this point. The two fish that did not exhibit a behavioural switch (both in UL treatment) followed relatively direct routes through the site. The majority of fish that responded did so in the intake channel (80% and 95% for UL and CH treatments, respectively). Four switches occurred more than 2 m from the intake channel so were deemed not to be influenced by hydrodynamic treatment and were therefore excluded from further analysis.

**Figure 4.**
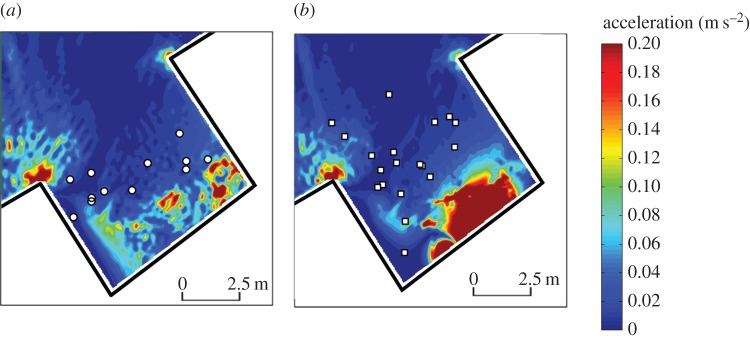
Locations of behavioural switch (*n* = 31) under two flow treatments: (*a*) unrestricted flow and low water acceleration (UL) and (*b*) constricted flow with high water acceleration (CH). Contour lines indicate velocity acceleration (m s^−2^).

In the CH treatment, switch points were distributed throughout the intake channel and area immediately upstream, whereas under the UL treatment they tended to be concentrated within a narrow band across the channel width (figure 4). Mean depth-averaged flow velocities at the points of behavioural switch ranged from 0.034 to 0.72 and from 0.14 to 0.67 m s^−1^ for UL and CH treatments, respectively. The median depth-averaged water velocity at the point of switch was higher in the UL compared with the CH treatment (0.67 and 0.57 m s^−1^, respectively; Mann–Whitney *U* = 2.68, *p* = 0.006). Velocity acceleration at the point of switch ranged from 0.001 to 0.051 and from 0.002 to 0.083 for UL and CH treatments, respectively, and did not vary among treatments (Mann–Whitney *U* = 1.28, *p* = 0.21).

**Figure 5.**
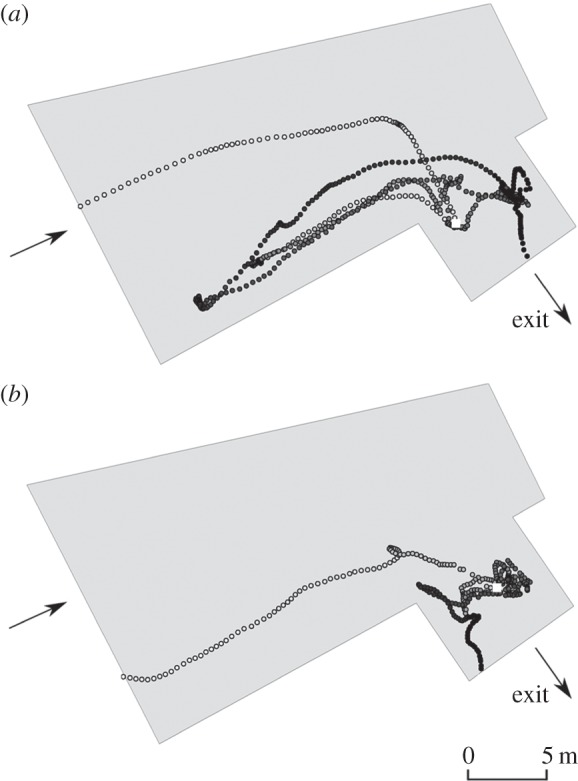
Example of trajectories of downstream-migrating adult eel through the forebay of a RHP facility. Tracks show (*a*) initial semi-passive drift followed by rejection in the intake channel, and (*b*) initial passive trajectory followed by exploratory behaviour. Point of behavioural switch is indicated by a white square. Flow direction is indicated by arrows.

Overall, rejection dominated behaviour immediately following a switch, apparent in 71% of fish. Treatment influenced post-switch behaviour as all fish exhibited rejection (figure 5*a*) under CH, whereas exploratory behaviour (figure 5*b*) was observed in 75% of individuals under UL treatment. Nearly all (91%) of the fish that rejected did so multiple times, up to a maximum of four occasions. Second points of rejection occurred closer to the bar rack than the first in 70% of cases.

Individuals in CH swam greater distances after behavioural switch than in the UL treatment (*t*_29_ = 2.23, *p* = 0.03, mean 89.3 ± 38.7 m and 54.3 ± 44.8 m (±s.d.), respectively), and a higher proportion of the post-switch tracks occurred outside of the intake channel (median proportion 68%, range 21–85%; Mann–Whitney *U* = 3.93, *p* < 0.001). Conversely, in the UL treatment, fish rarely left the intake channel after switch (median proportion 10%, range 0–68%). Mean eel ground speed (m s^−1^ over ground, unadjusted for water velocity) compared before and after a change in behaviour also revealed a treatment effect after switch (*t*_29_ = 2.88, *p* = 0.007), but not before (*t*_29_ = 1.67, *p* = 0.11). Ground speed was higher post-switch under CH (0.28 ± 0.05 ms^−1^, median ± s.d.) than for the UL treatment (0.23 ± 0.05 ms^−1^, median ± s.d.).

During downstream movements, 84% of eels were detected within 20 cm of the channel bed at the PIT antennae (I and II, figure 1), whereas upstream-moving fish were less frequently detected (56%), which may suggest a reduced tendency for benthic orientation after rejection.

## 4. Discussion

Manipulation of flow fields clearly influenced the behaviour of downstream-moving adult eels. On encountering flow acceleration eels displayed erratic behaviour, and the magnitude of response was positively related to maximum water velocity and flow acceleration. When flow was constricted, eels exhibited rejection as opposed to exploratory behaviour, which was observed predominantly under unrestricted conditions.

The response to the abrupt hydrodynamic transitions described may suggest the avoidance of hazardous areas that could cause damage or disorientation [25,33,35]. Other studies have demonstrated a rejection response exhibited by fish on encountering velocity gradients (e.g. juvenile Atlantic salmon [33] and juvenile Pacific salmon [34]). Although most eel rejections were several metres upstream of the bar rack, a small proportion (5%) could have been associated with physical contact at this structure. Findings differ from previous studies under both laboratory [51,52,60] and field conditions [46], which report that the majority of eels did not respond until making contact with structures. Although pre-contact rejection has been previously documented in the field [61,62], in this study both the close proximity to the intake at which the behavioural switch occurred and the positive relationship between magnitude of response and velocity acceleration provide evidence of the link between hydrodynamic stimuli and avoidance by eels.

In common with salmonids, eel behaviour during downstream migration was influenced by flow acceleration. To advance fish passage research, it is important that common concepts and approaches are adopted to aid the transfer of knowledge about different species and study systems [4,63]. A conceptual framework to understand and predict fish movement patterns in relation to complex flow fields around river structures is provided by Goodwin *et al.* [64]. In a model that describes four mutually exclusive downstream behavioural states (B1–4) used to govern the movements of simulated fish, individuals adjust swim orientation and speed in response to local water acceleration and pressure (depth). The first behaviour denotes fish movement with a biased correlated random walk, downstream in the direction of flow (B1). On approaching flow accelerations or decelerations, the fish exhibits one of two responses (determined by thresholds governed by recent past experience); either it orients swimming in the direction leading to faster water to facilitate obstacle and turbulence avoidance (B2), or a repulsive escape response is elicited in which the fish temporarily abandons downstream migration and swims upstream (B3). The fourth behaviour regulates fish response to pressure, and thus dictates swim depth. Incorporating all four behaviours provided the best fit between simulated fish and actual swim paths of juvenile salmonid smolts (*Oncorhynchus* sp.) descending complex flow fields at Lower Granite Dam, Snake River, USA [64].

The tendency for eels to align with streamlines in the upstream part of the forebay suggested advective behaviour (i.e. semi-passive drift with the local flow), which is broadly supported by the correspondence between eel ground speed and mean streamline velocity. Downstream movement was predominantly benthic-oriented, as observed in previous studies [45,46,48]. Therefore, the lower than 1 : 1 ratio of ground speed to water velocity probably reflects the lower velocities eels would have experienced near the channel bed, relative to modelled depth-averaged values. This resembled the B1 behaviour described by the Goodwin *et al.* [64] framework. However, in this study, the eels predominantly followed routes parallel to streamlines located close to the banks of the channel. Given their thigmotactic nature [51], it is surprising that individual eels seldom came into contact with the channel banks. Instead, our findings suggest that proximity to (rather than contact with) structures was used to some benefit. For eels, which undertake migration during dark and often turbid conditions which reduce visual cues, proximity to lateral boundaries may be an important navigational cue [65]. Fish are able to detect the hydraulic signatures created by structures through the mechanosensory system [23,30] and learn that near-field hydraulic patterns provide information on the environment beyond their sensory range [23]. For example, frictional resistance, resulting in decreasing average velocities towards the channel bed and banks, can be distinguished from form resistance induced by in-channel objects (e.g. rocks and woody debris), where water velocities increase due to reduced area, and increased travel distance, of flow around the object [23,24]. It was not clear what hydraulic signatures were detected by eels to identify the lateral banks. There was no apparent difference in characteristics between the streamlines eels descended, near the lateral boundaries, and those in the centre of the channel. It is plausible that such hydraulic signatures could be identified by using three-dimensional numerical models because they provide information on flow features induced by lateral boundaries (e.g. secondary currents) that cannot be captured by two-dimensional models such as the one used in this study.

The response of eels on encountering velocity acceleration depended on both the novelty and strength of the transition stimuli. When approaching rapid velocity acceleration, most fish responded by rejecting upstream, a behaviour that closely corresponded to B3 in the Goodwin *et al.* framework [64]. This is postulated to occur when the relative change in acceleration exceeds a threshold intensity, causing the fish to swim in the opposite direction to the principal velocity vector and return upstream [64]. The greater the magnitude of acceleration, the faster the subsequent swim speed during eel response. A similar relationship has been observed for salmon smolts rejecting decelerating flow [28]. When approaching a less abrupt acceleration transition, eels ventured closer to the intake, and therefore experienced higher velocities before switching to slower exploratory behaviour. This broadly conforms to similar milling/exploratory behaviour in salmonids [66], expressed as recursive cycles between behaviours B3 and B1–2 in modelled fish [64]. Eels are influenced more by thigmotactic cues than salmonids [67], thus observed exploratory behaviour may have been both hydraulically and thigmotactically mediated. The propensity of eels to explore their environment has been associated with active searching for a way through (or alternative route past) screens and bar racks at hydropower and pumping facilities [46,61]. In this study, eels generally restricted exploration to the area within the intake channel, yet were rarely detected to contact the bar rack.

After their first response to velocity acceleration in the intake channel, individuals appeared to become somewhat habituated to the transition and more likely to pass through the same region on subsequent encounters. For a fish to detect change in a stimulus relative to the background noise it is acclimated to, the stimulus must exceed a threshold value (termed ‘just notable difference’). Therefore, the response is dependent on exposure history [24,68]. Such adaptive behaviour enables animals to repeatedly test their environment and adjust their risk of exposure to potentially harmful elements based on prior experience.

The importance of hydrodynamics in influencing eel behaviour has significant implications for progressing guidance and passage technologies for this threatened species. Eel bypasses should be designed to avoid abrupt velocity acceleration at the entrance, as is currently advised for salmonids [15,69], with the aim to minimize rejection. Conversely, avoidance behaviours present an opportunity to guide eels away from dangerous areas and towards safe passage routes. There is clear potential for hydrodynamic-based guidance to enhance the effectiveness of traditional physical screens that can be expensive to install and maintain, reduce power generation or pumping efficiency, and may still induce fish damage and mortality through collision and impingement [37,43]. Indeed, flow manipulation to guide downstream migrants past river infrastructure has been applied with some success for juvenile salmonids [70,71], and may have value for eels too. All individuals that rejected ultimately habituated to intake conditions and passed, highlighting that the response to acceleration fields is adaptive. Eels have also been shown to quickly habituate after initial rejection induced by water jets and air bubbles [52]. Accordingly, effective behavioural guidance devices must efficiently divert fish to alternative routes (e.g. a bypass) prior to habituation.

Semi-passive drift probably accounts for the majority of downstream adult silver eel movement through lotic systems, though it was apparent that they reject abrupt changes in flow fields on the approach to structures and explore upstream until continuing their migration. Rapid acceleration triggered upstream rejection, whereas less abrupt acceleration caused slower, exploratory behaviour. The increased resolution afforded by the fish-positioning telemetry and flow-mapping techniques employed in this study has challenged historical perceptions about eel behaviour derived from more coarse-scale investigations. These advances represent an important step forward in the drive to develop effective guidance and passage solutions for this species at anthropogenic barriers. Combining fine-scale fish movement data with empirically informed hydrodynamic models offers great potential to further our limited understanding of fish behaviour in relation to the complex hydrodynamic environments encountered at river infrastructure.

## Data accessibility

Fish trajectory data file available at Dryad: doi:10.5061/dryad.c77jn.
